# No Confirmed Cases of *Taenia solium* Taeniasis in a Group of Recently Arrived Sub-Saharan Migrants to Italy

**DOI:** 10.3390/pathogens8040296

**Published:** 2019-12-14

**Authors:** Lorenzo Zammarchi, Marta Tilli, Antonia Mantella, Annarita Botta, Alessandra Nicoletti, Héctor Hugo García, Yesenia Castillo, Donatella Aquilini, Sara Boccalini, Alessandro Bartoloni

**Affiliations:** 1 Department of Experimental and Clinical Medicine, University of Florence, 50134 Florence, Italy; marta.tilli@unifi.it (M.T.); antonia.mantella@unifi.it (A.M.); annarita.botta@unifi.it (A.B.); alessandro.bartoloni@unifi.it (A.B.); 2 Infectious and Tropical Diseases Unit, Careggi University and Hospital, 50134 Florence, Italy; 3 Referral Center for Tropical Diseases of Tuscany Region, Careggi University Hospital, 50134 Florence, Italy; 4 Department “GF Ingrassia”, Section Neuroscience, University of Catania, 95123 Catania, Italy; anicolet@unict.it; 5 Department of Microbiology and Center for Global Health—Tumbes, Universidad Peruana Cayetano Heredia, Lima 15102, Peru; hgarcia1@jhu.edu (H.H.G.); yesenia.castillo.b@upch.pe (Y.C.); 6 Cysticercosis Unit, National Institute of Neurological Sciences, Lima 15102, Peru; 7 Infectious Diseases Unit, Hospital of Prato, 59100 Prato, Italy; donatella.aquilini@uslcentro.toscana.it; 8 Department of Health Sciences, University of Florence, 50134 Florence, Italy; sara.boccalini@unifi.it

**Keywords:** *Taenia solium*, Sub-Saharan Africa, taeniasis, cysticercosis, neurocysticercosis, migrants

## Abstract

One-hundred and sixty-four migrants from Sub-Saharan Africa to Italy were screened with the *Taenia solium* specific enzyme-linked immunosorbent assay coproantigen (ELISA CoAg) and four (2.4%) were recorded as positive, but with optical density values near to the cut-off. No ELISA CoAg positive samples were confirmed by parasitological methods. Low positivity could be attributed to false positive result or cross-reaction with other *Taenia* species. Further studies are needed to assess the role of migration on sporadic autochthonous transmission of *T. solium* taeniasis/cysticercosis in Europe.

## 1. Introduction

*Taenia solium* taeniasis/cysticercosis is a public health concern in several middle- and low-income countries, including countries in the region of Sub-Saharan Africa (SSA), where the prevalence of tapeworm carriers ranges from 1.5% to 5% [1], and neurocysticercosis is responsible of 30% of cases of epilepsy [2]. Traditional parasitological examination of stool has a low sensitivity, ranging from 0% to 63% [3], and is not usually able to differentiate human tapeworm species, since the eggs of *T. solium, T. saginata* and *T. asiatica* are morphologically indistinguishable and the differentiation based on scolices, which are rarely recovered intact, and mature proglottids is not always feasible [4]. The species-specific enzyme-linked immunosorbent assay (ELISA) coproantigen (CoAg) for the diagnosis of *T. solium* taeniasis has shown a higher sensitivity (81–100%) and specificity (99%) [3].

The complete life cycle of *T. solium* taeniasis/cysticercosis probably no longer occurs in Italy, since the last autochthonous *T. solium* taeniasis case diagnosed in Italy was recorded in Sicily in 1985, and cases of porcine cysticercosis were not detected in a survey carried out in 2007 in Italy [5,6,7].

However, migration from endemic countries may theoretically ignite reintroduction of the parasite and sporadic autochthonous transmission in receiving countries. *T. solium* tapeworm carriers can cause foci of neurocysticercosis, even among contacts who do not eat pork and have not traveled to a country where the parasite is endemic [8]. A recent review has identified 78 cases of United States (US)-acquired cysticercosis, in both foreign and US-born populations [9]. Migration flows from SSA to Europe were particularly intense in recent years. In the period of 2014–2019, 650,000 migrants disembarked in Italy, the majority from SSA [10]. In 2014, 274 asylum seekers hosted in a large reception center in Sicily were tested with the parasitological examination of stool and six (2.16%), all from the Horn of Africa (five from Eritrea and one from Ethiopia, respectively), were found positive for *Taenia* spp. eggs. Data on *Taenia* species were not available in this study [11]. We report the results of a study aimed at investigating the prevalence of *T. solium* taeniasis in a population of SSA migrants recently arrived in Italy using the species-specific ELISA coproantigen (CoAg).

## 2. Results

Six-hundred and thirty-nine people participated in health information meetings, 40% (254/639) agreed to participate in the study, and 64% (164/254) provided a stool sample. All the participants to this study were males. The median residency time in Italy of subjects providing the stool sample was 28 months (range 7–36), and the median age was 24 years (range 18–49). The most frequent country of origin was Nigeria (69/164, 42%), followed by Guinea (22/164, 13%) and Ivory Coast (16/164, 10%). Twenty-seven (16%) reported consumption of pork, 42 (25%) denied it, and the remaining preferred not to respond to this question.

The ELISA-CoAg was reactive on four (2.4%, 95% CI 0.1–4.7%) subjects. In three cases, the ratio between Optical Density of the sample (ODs) and Optical Density of the positive pool (ODpp) (ODs/ODpp) showed values near to the cut-off (borderline). No *Taenia* egg was detected in stool samples by microscopy. Positivity was not associated with gastrointestinal or neurological symptoms. The characteristics of subjects with a ELISA-CoAg reactive test are reported in Table 1. Interestingly, of the five Somali men enrolled in the study, two (40%) had a positive ELISA CoAg [11]. Only one patient was available for follow-up, while the remaining were lost to follow-up. The patient received treatment with niclosamide (2 g orally repeated after one week) and was asked to bring stools obtained over three days after the first niclosamide dose. The patient was instructed on the possible emission of a tapeworm with his stool after the treatment. Iconographic material was used to show him the macroscopic appearance of the worm, in a similar way to what was described by Flisser and colleagues in their studies on self-diagnosis of taeniasis [12]. Only one stool sample was provided and no proglottid or scolex were detected. The patient was contacted by telephone and denied having eliminated any tapeworm. The enrollment was interrupted before the estimated sample size could be reached, following a quick reduction in the number of migrants arriving in Italy in recent years due to the changed geopolitical scenario.

## 3. Discussion

Our findings do not confirm the presence of *T. solium* tapeworm carriers among recently arrived migrants from SSA to Italy, since no reactive ELISA CoAg samples were confirmed by parasitological methods and in three cases, ODs/ODpp showed values near to the cut-off. In this scenario, it is impossible to determine if there was a *T. solium* infection. Usually, confirmed positive samples present much higher values and weak positivity may be linked to false positive results or a cross-reaction with other *Taenia* species. However, we cannot completely exclude the presence of *T. solium* infection based on a negative microscopic examination of stool only, since this test has a low sensitivity. Furthermore, since the treatment rate of niclosamide is not 100% [13], the absence of recovered scolex/proglottids after treatment do not necessarily indicate that the treated patient was not infected. Moreover, only one follow-up stool sample was tested. A low prevalence may be also explained by the fact that subjects enrolled in this study were tested with a median time of 28 months from their arrival in Italy and *T. solium* tapeworms are believed to live around three years [14], so it is possible that the parasite died before our sampling. It must be noted that the majority of enrolled subjects come from countries where the most common religion is Islam, which prohibits pork consumption. However, the same amount of time between migration and testing, and almost the same origin/religion distribution, was also present in the previous study on a similar population conducted by Patamia et al., which found a higher prevalence of *Taenia* spp. at microscopy (2.16%) [11]. Further studies on migrant population, based on a sensitive and specific diagnostic test for *T. solium,* such as the CoAg on a larger sample with parasitological or molecular confirmation of positive samples, are needed to assess the possible reemergence or sporadic autochthonous transmission of *T. solium* taeniasis/cysticercosis in nonendemic European countries.

## 4. Materials and Methods

Between March 2018 and July 2019, a prevalence study on *T. solium* taeniasis was carried out on SSA migrants hosted in 12 reception centers for male subjects in five health districts of the province of Florence and Prato, Tuscany Region, Italy.

The study was proposed on a voluntary basis to approximately 700 SSA adult migrants. Inclusion criteria were being aged ≥ 18 years and arrived in Italy for less than three years before the enrollment date. The exclusion criterion was a previous treatment for any parasitosis. A sample size of 640 subjects was required to allow an estimation of the prevalence of *T. solium* taeniasis with a confidence interval of 90%, with a margin of error of 2.5% and an expected frequency of 1.5%. The study was approved by the ethics committee of Area Vasta Centro on 16/01/2018 (code 12298_bio). Migrants were invited to participate to the study during short information events on health topics (such as infectious diseases prevention) delivered in English and French, with the help of cultural mediators when needed. After having signed the informed consent, subjects willing to participate were asked to answer to a short questionnaire to collect epidemiological and clinical information and to provide a stool sample for direct parasitological examination and *T. solium* ELISA CoAg. Stool samples were collected and preserved within four hours from the time of collection by homogenization in phosphate-buffered saline and formaldehyde at 5% (10 g of stool with 40 ml, 1:4), and later sent to the Cysticercosis Unit, Universidad Peruana Cayetano Heredia, Lima, Peru, for direct parasitological examination and *T. solium* ELISA CoAg. The latter was performed according to Allan et al. [15] and considered reactive if the ODs/ODpp was higher than 7.5.

## Figures and Tables

**Table 1:** Characteristics of patients with reactive *Taenia solium* enzyme-linked immunosorbent assay coproantigen.

Age	Country of Origin	Months of Residence in Italy	(OD Sample/ OD Positive Pool) × 100	Pork Consumption	Stool Microscopy	Treatment with Niclosamide	Follow-Up after Treatment
23	Guinea	35	16.733	No	Neg	Yes	No proglottids/scolices detected in follow-up stool sample
37	Somalia	24	8.904	No	Neg	No	Lost to follow-up
33	Somalia	22	7.949	No	Neg	No	Lost to follow-up
27	Cote d’Ivoire	20	9.332	No	Neg	No	Lost to follow-up
